# Preparation of umami octopeptide with recombined *Escherichia coli*: Feasibility and challenges

**DOI:** 10.1080/21655979.2017.1378839

**Published:** 2017-09-28

**Authors:** Liming Zhao, Yin Zhang, Chandrasekar Venkitasamy, Zhongli Pan, Longyi Zhang, Siya Guo, Wei Xiong, Hu Xia, Liu Wenlong, Gou Xinhua

**Affiliations:** a Key Laboratory of Meat Processing of Sichuan, Chengdu University, Chengdu, China; b State Key Laboratory of Bioreactor Engineering, R&D Center of Separation and Extraction Technology in Fermentation Industry, East China University of Science and Technology, Shanghai, China; c Department of Biological and Agricultural Engineering, University of California, Davis, Davis, CA, USA; d Healthy Processed Foods Research Unit, Western Regional Research Center, USDA-ARS, Albany, CA, USA

**Keywords:** fusion protein, octopeptide, recombinant bacteria, umami, umami peptide

## Abstract

The taste of umami peptide H-Lys-Gly-Asp-Glu-Glu-Ser-Leu-Ala-OH (LGAGGSLA) is controversial. One possible reason for this controversy is the use of chemically synthesized LGAGGSLA to confirm its taste. To explore other ways to further confirm the flavor of LGAGGSLA, we developed a new strategy to prepare a bio-source peptide by adopting a gene engineering method to express LGAGGSLA in recombinant *Escherichia coli*. In our previous work, we structured the LGAGGSLA recombinant expression system and optimized the culturing conditions for preparing a fusion protein. However, the fusion protein was not cleaved by enterokinase to obtain LGAGGSLA. Because the cleavage conditions of commercial enterokinase were not specific and recombinant engineered bacteria had the potential to be used in industrial processes, in this addendum, we calculated the mass and volume yields of key processing steps in the preparation of LGAGGSLA, and established a model of cleavage conditions with the cleavage ratio of LGAGGSLA. When the LGAGGSLA was confirmed to show umami taste, it is considered as a new umami or umami enhancer. The gene information of LGAGGSLA should have a great potential in the development of new flavor product and food product containing high umami flavor.

## Introduction

In our recent work, we structured the umami recombinant expression of octopeptide in *Escherichia coli*, H-Lys-Gly-Asp-Glu-Glu-Ser-Leu-Ala-OH (LGAGGSLA), and optimized its culturing conditions for preparing a fusion protein.^1^ It was reported that LGAGGSLA showed umami taste in 1978,^2^ but its flavor was controversial due to the inconsistency in the verification results.^3^ Use of synthesized LGAGGSLA to confirm its taste might be the most likely reason for the flavor disputation.^3-5^ Based on this speculation, we tried to prepare bio-source LGAGGSLA to further confirm its taste. However, we just obtained the fusion protein in our previous work, which had to be cleaved by enterokinase (EK) to obtain LGAGGSLA.

Cleavage efficiency and the purification process are important in obtaining the target peptides.^6^ The major factors influencing the cleavage efficiency and target peptide purification are EK dosage, time, temperature, and pH. Shahravan, et al.^7^ investigated the effect of the amount of EK, pH, and temperature on the yield of desired protein products from EK proteolysis. It was found that EK dosage showed no effect on cleavage pattern for the aggregation of fusion protein. Cui, et al.^8^ found hydrogen peroxide could accelerate EK cleaving fusion proteins at low temperature. To use EK economically, Kubitzki^9^ developed a feasible process by immobilizing EK on a porous material to cleave fusion proteins. There have not been many investigations on the relationship between cleavage conditions and the cleavage ratio of target peptides.

The recommended cleavage conditions of commercial EK were very general. To obtain the optimal cleavage conditions, the optimization experiments should be performed. Therefore, we set up a model of hydrolysis conditions with the cleavage ratio of LGAGGSLA. Since structured engineering bacteria can be adopted to prepare LGAGGSLA in laboratories and also have potential to be used in industrial processes, the mass and volume yields of key steps in the process of preparing LGAGGSLA were calculated in this addendum.

### Flow chart of preparing bio-source umami octopeptide

**Figure 1.**
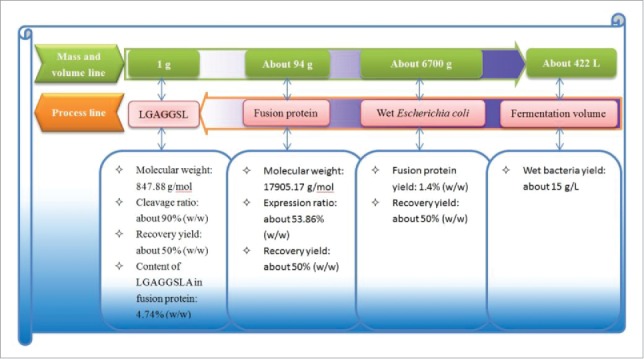
Flow chart of preparing bio-source umami octopeptide.

To demonstrate the process of preparing LGAGGSLA, a process flow chart and the corresponding mass and volume yields of key steps are shown in Fig. 1. There are 5 key steps in the process of preparing LGAGGSLA. The first step is to inoculate the recombinant *E.coli* to Luria- Bertani (LB) medium to ferment; the second step is to centrifuge the culture to obtain the wet *E.coli*; the third step is to break the bacteria and purify the supernatant to obtain a fusion protein. The last step is to cleave the fusion protein with EK and purify the hydrolysate to produce LGAGGSLA.

The mass and volume yields for each key unit are shown in Fig. 1. The data indicate that it needs about 94 grams of fusion protein to produce 1 gram of LGAGGSLA based on the cleavage ratio (about 90%), recovery yield (about 50%), and content of LGAGGSLA in the fusion protein (4.74%). To prepare the 94 grams of fusion protein, about 6700 grams of wet *E.coli* are needed when the fusion protein yield is 1.4% and recovery yield is about 50% of purifying fusion protein. Given the wet *E.coli* yield of fermentation is about 15 g/L, about 448 L fermentation broth is needed.

To evaluate the cost benefit of preparing LGAGGSLA, its cost was compared with that of preparing typical umami flavors, such as monosodium glutamate or chicken essence. The price of LGAGGSLA is about 7000 dollars per ton, while the prices of the monosodium glutamate and chicken essence are about 1200 dollars per ton and 1000 dollars per ton, respectively. The cost of LGAGGSLA is about 7 times that of the monosodium glutamate or chicken essence. Therefore, using present recombinant bacteria to prepare LGAGGSLA is not economically viable for industrial production. However, the gene information of LGAGGSLA can be used to develop manmade food products if its flavor is confirmed, such as in vitro manmade meat using cell-culture technology.^10^

### Fitting model of preparing umami octopeptide by EK hydrolysis

Since the cost of preparing LGAGGSLA is high, it is necessary to optimize cleavage conditions to increase cleavage efficiency. A response surface method was adopted to design the optimization experiments of the cleavage conditions. Based on the fitted results, one model of correlating the cleavage ratio of LGAGGSLA and cleavage conditions (EK dosage (μL/mg), cleavage time (h), and cleavage temperature (°C)) was established (formula 1). According to formula 1, the optimal cleavage conditions were EK dosage of 2.00 μL/mg, cleavage time of 15 h, and cleavage temperature of 24°C. The maximum cleavage ratio of LGAGGSLA was 93.1%.

Formula 1 can also be used to predict any one of Y, X1, X2, and X3 if three of them are known, which is useful for changing cleavage conditions. Taking the X1 (EK dosage) as an example, since the price of EK is expensive, to reduce its dosage, the minimum value of X1 can be calculated if the value of Y (cleavage ratio of LGAGGSLA), X2 (cleavage time) and X3 (cleavage temperature) are known.

(formula 1) $$\begin{array}{l} \text{Y =}\text{−69}\text{.7907+48}\text{.46178*X1+10}\text{.38159*X2}\\ \text{+2.085563*X3}\text{−7}\text{.279636*X1*X1}\\ \text{−0.798681*X1*X2}\text{−0}\text{.134434*X1*X3}\\ \text{−0.249185*X2*X2−0}\text{.028895*X2*X3}\\ \text{−0.023846*X3*X3 } \end{array}$$

where, Y is the cleavage ratio of LGAGGSLA and X1, X2 and X3 represent EK dosage, cleavage time, and cleavage temperature, respectively.

### Feasibility and challenges

The present results of recombination of *E. coli*, purification of fusion protein, and cleavage of LGAGGSLA with EK indicate that it is feasible to use the gene engineering method to prepare bio-source LGAGGSLA. However, more challenges still remain. The first is that the content of LGAGGSLA in the fusion protein is not high, making it difficult to obtain a large amount of target peptide in a short period of time and with low cost. The second challenge is that as the EK is not a sequence specific cleavage enzyme, it often cleaves at non- target sites.^11^ This makes the purification process complicated, resulting in a low recovery yield of LGAGGSLA. Therefore, a more efficient expression system of LGAGGSLA should be restructured if its taste is confirmed. The structured bacteria should yield more LGAGGSLA and be suitable for producing large amounts of LGAGGSLA through industrial fermentation.

## Conclusions

Based on our previous investigation of structuring recombinant *Escherichia coli* for preparing LGAGGSLA, the mass and volume yields of key processing steps in the preparation of LGAGGSLA were calculated, and the cleavage conditions of using EK to hydrolyze the fusion protein were optimized with response surface method in this addendum. The results showed that using present recombinant bacteria to prepare LGAGGSLA is not economically viable for industrial production. The obtained model of cleavage conditions with the cleavage ratio of LGAGGSLA can be used to predict the ratio or processing conditions. The optimal cleavage conditions of preparing LGAGGSLA were EK dosage of 2.00 μL/mg, cleavage time of 15 h, and cleavage temperature of 24°C, with corresponding the maximum cleavage ratio of LGAGGSLA as 93.1%.

## Abbreviations

LGAGGSLA: H-Lys-Gly-Asp-Glu-Glu-Ser-Leu-Ala-OH
LB: Luria- Bertani
EK: enterokinase
